# Prognostic analysis of cT1-3N1M0 breast cancer patients who have responded to neoadjuvant therapy undergoing various axillary surgery and breast surgery based on propensity score matching and competitive risk model

**DOI:** 10.3389/fonc.2024.1319981

**Published:** 2024-01-24

**Authors:** Maoquan Zhang, Yingming Sun, Huasheng Wu, Jian Xiao, Wenxin Chen, Hebin Wang, Binglin Yang, Huatian Luo

**Affiliations:** ^1^ Department of Breast Surgery, Affiliated Sanming First Hospital of Fujian Medical University, Sanming, Fujian, China; ^2^ Department of Medical and Radiation Oncology, Affiliated Sanming First Hospital of Fujian Medical University, Sanming, Fujian, China; ^3^ Department of Hepatobiliary Surgery, Affiliated Sanming First Hospital of Fujian Medical University, Sanming, Fujian, China

**Keywords:** neoadjuvant therapy, sentinel lymph node biopsy, breast-conserving surgery, propensity score matching, SEER database

## Abstract

**Background:**

Sentinel lymph node biopsy (SLNB) in breast cancer patients with positive clinical axillary lymph nodes (cN1+) remains a topic of controversy. The aim of this study is to assess the influence of various axillary and breast surgery approaches on the survival of cN1+ breast cancer patients who have responded positively to neoadjuvant therapy (NAT).

**Methods:**

Patients diagnosed with pathologically confirmed invasive ductal carcinoma of breast between 2010 and 2020 were identified from the Surveillance, Epidemiology, and End Results (SEER) database. To mitigate confounding bias, propensity score matching (PSM) analysis was employed. Prognostic factors for both overall survival (OS) and breast cancer-specific survival (BCSS) were evaluated through COX regression risk analysis. Survival curves were generated using the Kaplan-Meier method. Furthermore, cumulative incidence and independent prognostic factors were assessed using a competing risk model.

**Results:**

The PSM analysis matched 4,890 patients. Overall survival (OS) and BCSS were slightly worse in the axillary lymph node dissection (ALND) group (HR = 1.10, 95% CI 0.91-1.31, p = 0.322 vs. HR = 1.06, 95% CI 0.87-1.29, p = 0.545). The mastectomy (MAST) group exhibited significantly worse OS and BCSS outcomes (HR = 1.25, 95% CI 1.04-1.50, p = 0.018 vs. HR = 1.37, 95% CI 1.12-1.68, p = 0.002). The combination of different axillary and breast surgery did not significantly affect OS (p = 0.083) but did have a significant impact on BCSS (p = 0.019). Competing risk model analysis revealed no significant difference in the cumulative incidence of breast cancer-specific death (BCSD) in the axillary surgery group (Grey’s test, p = 0.232), but it showed a higher cumulative incidence of BCSD in the MAST group (Grey’s test, p = 0.001). Multivariate analysis demonstrated that age ≥ 70 years, black race, T3 stage, ER-negative expression, HER2-negative expression, and MAST were independent prognostic risk factors for both OS and BCSS (all p < 0.05).

**Conclusion:**

For cN1+ breast cancer patients who respond positive to NAT, the optimal surgical approach is combining breast-conserving surgery (BCS) with SLNB. This procedure improves quality of life and long-term survival outcomes.

## Introduction

To assess the prognosis of breast cancer patients and guide their treatment, it is crucial to determine the status of axillary lymph nodes (ALN). For patients with early-stage breast cancer who have negative ALN and present clinically low risk, guidelines recommend the use of sentinel lymph node biopsy (SLNB) (1–3). When sentinel lymph node (SLN) shows no evidence of tumor, axillary lymph node dissection (ALND) can be omitted, streamlining surgical procedures, reducing hospitalization duration, and minimizing complications like upper limb lymphedema and dysfunction, all without compromising survival (4, 5). In patients with early-stage breast cancer where ALN are negative and clinical risk is low, even if SLN indicates the presence of 1 or 2 macro metastases, ALND can still be avoided by opting for breast-conserving surgery (BCS) combined with radiotherapy (RT) (5).

In order to preserve both the axillary and breast regions, neoadjuvant therapy (NAT) is typically administered as the initial treatment for cN1+ breast cancer, particularly in patients with HER-2-positive breast cancer and triple-negative breast cancer. Concurrently, the use of precise *in vivo* drug sensitivity testing can identify high-risk groups for escalated treatment, ultimately enhancing patient prognosis (1, 2, 6–10). After receiving NAT, the percentage of breast cancer patients with clinically positive lymph nodes (cN1+) that transitioned to clinically negative lymph nodes (cN0) was as high as 46% to 91%. Consideration of SLNB is warranted if positively identified nodes with a locator clip are excised during the operation, or if SLN is identified using a dual tracer and at least three SLN are detected. With negative test results, 30.3% to 56.5% of patients can avoid ALND (7, 11–18).

However, cN1+ patients who respond positive to NAT may face challenges in preserving both the breast and axillary regions due to various factors (12, 19–24). Firstly, several factors can obstruct lymphatic drainage in the breast, affecting the detection of SLN, such as tumor cell necrosis, non-bacterial inflammation, and lymphatic fibrosis. Second, tumor regression may occur in an irregular pattern, resulting in unacceptable false-negative and margin-positive rates. Additionally, a higher false-negative rate (8.4%-17%) is observed in patients who do not use dual tracers or marker clips to locate the SLN. Lastly, there is a lack of robust long-term survival data. While SLNB is performed for cN1+ breast cancer patients who respond positive to NAT, if the SLNs are positive, the standard treatment still involves supplementary ALND and local RT (1, 2, 6).

Although cN1+ patients who respond positive to NAT may encounter various challenges, including different degrees of false negative rates, performing SLNB remains an acceptable approach to avoid ALND (1, 2, 6, 11–17, 19, 20). However, it’s worth noting that the majority of studies in this area are non-randomized, single-center, and characterized by small sample sizes, limited biopsy techniques, short follow-up periods, and a lack of long-term survival data. Consequently, the experimental conclusions need further validation. The SEER program, hosted by the National Cancer Institute, encompasses nearly half of the U.S. population and provides invaluable research data for the prevention and management of cancer patients. In light of this, the present study retrospectively analyzed patients with cT1-3N1M0 breast non-specific infiltrating duct carcinoma who responded positive to NAT between 2010 and 2020 in the SEER database. The objective was to investigate the impact of various axillary and breast surgical approaches on survival, thereby furnishing critical clinical evidence for the reasonable selection of axillary and breast surgery.

## Materials and methods

### Data collection

In this study, the SEER database data were obtained by searching the SEER database [Incidence-SEER Research Data, 17 Registries, Nov 2022 Sub (2000–2020)] with software SEER*Stat v8.4.1.2 (download from https://seer.cancer.gov/data-software/) and account numbers (access code is: #89bMxdH, obtained from https://seer.cancer.gov/data/access.html). The SEER data obtained did not contain any personally identifiable patient information. As a result, this study was exempt from ethical review by the Ethics Committee of the Affiliated Sanming First Hospital of Fujian Medical University.

### Patient cohort

**Figure 1:**
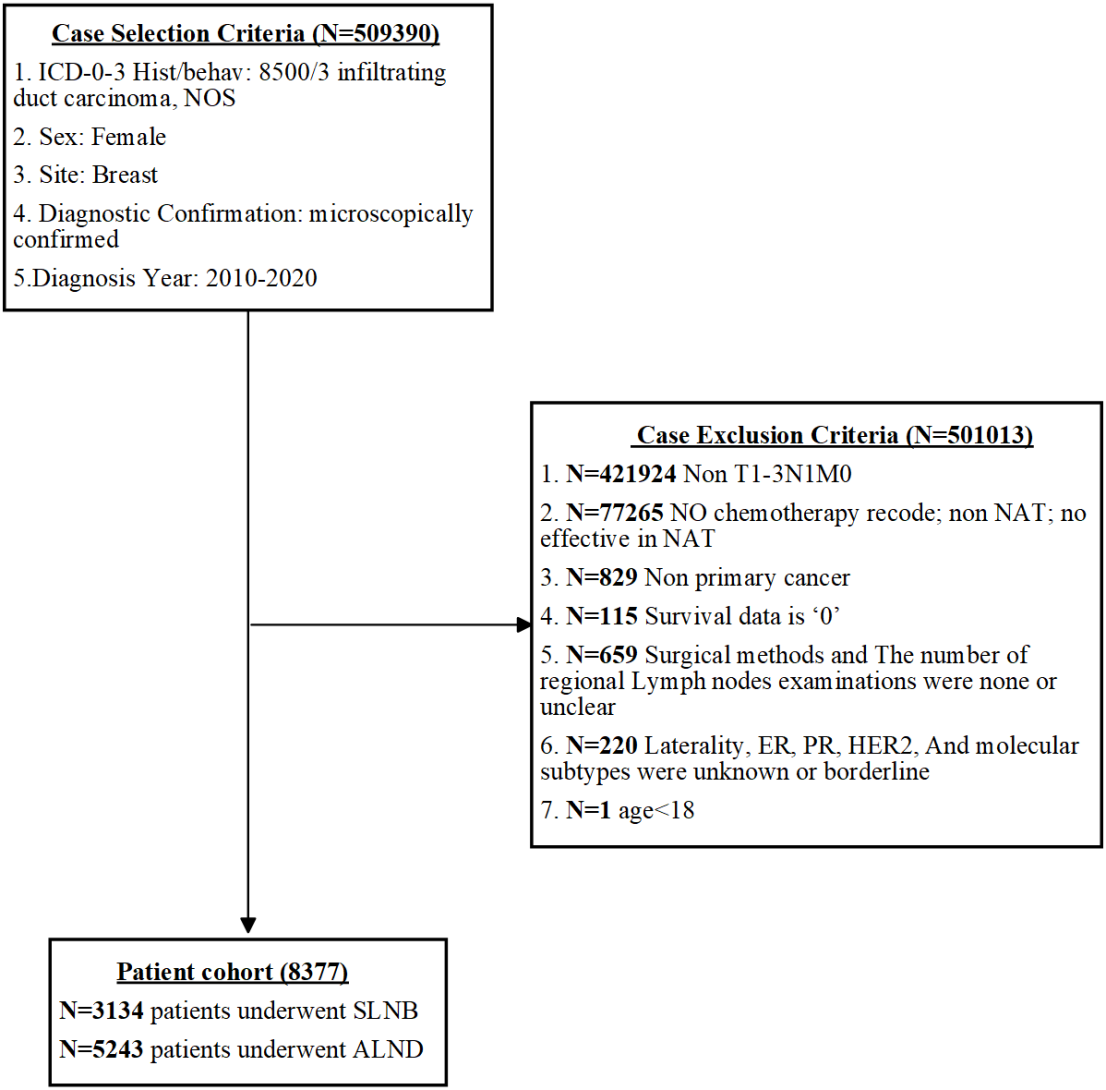
The screening process of the patient cohort in this study.

Patients included in this study were females with a confirmed pathological diagnosis of nonspecific infiltrating duct carcinoma of the breast (ICD-0-3 = 8500/3) from 2010 to 2020. The collected data encompassed various factors such as age, marital status, race, laterality, histological grade, TNM classification, molecular subtypes, primary cancer details, records of radiotherapy and chemotherapy, surgical records, count of regional lymph node examinations, treatment sequencing, follow-up duration, survival status, and cause of death. In accordance with AJCC 8th edition guidelines, data for T1, T2, T3, and N1 patients were integrated from 2010 to 2020, and M0(i+) was considered as M0. The exclusion criteria consisted of the following: [1] absence of chemotherapy records, non-NAT, and ineffectiveness in NAT; [2] non-primary cancer; [3] survival data is 0; [4] unknown surgical methods and count of regional lymph node examinations; [5] indeterminate or missing information regarding laterality, ER, PR, HER2, and molecular subtypes; and [6] age < 18. **Figure 1** illustrates the detailed design process of this study.

The age variable was stratified into four groups based on the onset of breast cancer: < 35, 35-54, 55-69, and ≥ 70. Marital status was categorized into three groups: married, single, and other. Race was divided into three groups: white, black, and other. Due to a substantial amount of data with unknown histological grading, this subset was retained and treated as a separate variable, further divided into three groups: grade I-II, grade III-IV, and unknown. Given that the SEER database did not distinguish between specific axillary procedures, making it difficult to differentiate between SLNB and ALND, this study followed the axillary dissection definition for breast cancer as outlined by AJCC and supported by relevant literature (25, 26). In this study, regional lymph node detection numbering between 1-5 was classified as SLNB, while detection of 6 or more nodes was classified as ALND. Additionally, following guidelines provided by the SEER database Breast Surgery Code Manual, codes 20-24 were identified as indicative of BCS for breast cancer, whereas codes 30 and 40-75 were associated with MAST procedures for breast cancer. In order to provide more tailored guidance for clinical practice, the study conducted a survival analysis of combined axillary and breast surgeries (BCS+SLNB, BCS+ALND, MAST+SLNB, and MAST+ALND).

### Observation indicators

The observational analysis in this study focused on several key indicators, including overall survival (OS), breast cancer-specific survival (BCSS), breast cancer-specific death (BCSD), and death from other causes (OCSD). OS was defined as the duration from diagnosis to either death from any cause or the last follow-up. BCSS and BCSD measured the period from diagnosis to death attributed specifically to breast cancer or until the last follow-up. OCSD denoted the interval between diagnosis and death resulting from reasons other than breast cancer.

### Statistical analysis

In this study, all variables were categorical and expressed as percentages. Chi-square tests were employed to assess differences between groups of variables. Propensity score matching (PSM) analysis was conducted using the R package “MatchIt”. The nearest neighbor matching algorithm was implemented with a matching ratio of 1:1 and a caliper value of 0.001. This aimed to balance variables that exhibited significant differences between the SLNB group and the ALND group, thereby reducing potential confounding biases in this retrospective study. Kaplan-Meier survival analysis, facilitated by the R packages “survival” and “Survminer”, was utilized to estimate survival probabilities and generate survival curves. Inter-group comparisons were conducted using the log-rank test. Univariate and multivariate Cox proportional hazard regression models were applied to analyze independent prognostic risk factors for OS and BCSS, with results presented in forest plots. The R package “cmprsk” was utilized for competing risk model analysis to mitigate estimation bias related to deaths from other causes. The Fine-Gray test was employed to obtain cumulative incidence data for different axillary and breast surgeries. A multivariate analysis of the competitive risk model was performed using the R package “mstate”. This facilitated the construction of a COX regression model and the creation of a nomogram. All statistical analyses were conducted using R Studio (R 2023.06.0 + 421, downloaded from https://posit.co/downloads/).A significance level of *p* < 0.05 was considered statistically meaningful.

## Results

### Patient clinicopathological characteristics

Before propensity score matching (PSM), a total of 8,377 eligible breast cancer patients were included, with 3,134 in the SLNB group and 5,243 in the ALND group. In comparison to the ALND group, the SLNB group exhibited higher incidences of left breast tumors (52.3%), unknown histological grade (58.0%), T1 staging (25.5%), ER-negative expression (39.9%), PR-negative expression (53.7%), HER2 positive expression (40.7%), HR+/HER2+ subtype (27.0%), and a higher proportion of BCS (48.8%), with all differences being statistically significant (all *p* < 0.05). After PSM, a total of 4,890 eligible breast cancer patients were included, with 2,445 in the SLNB group and 2,445 in the ALND group. After matching, there were no statistically significant differences between the two groups across all variables (all *p* > 0.05). This indicates a successful matching outcome. Detailed baseline characteristics of patients before and after PSM are presented in **Table 1**.

**Table 1:** The clinicopathological characteristics of patients before and after PSM.

Characteristics	Before PSM				After PSM			
	All patients (n=8377) N(%)	SLNB (n=3134) N(%)	ALND (n=5243) N(%)	P value	All patients (n=4890) N(%)	SLNB (n=2445) N(%)	ALND (n=2445) N(%)	P value
Age				0.134				0.349
<35	651 (7.8)	248 (7.9)	403 (7.7)		342 (7.0)	175 (7.2)	167 (6.8)	
35-54	4496 (53.7)	1631 (52.0)	2865 (54.6)		2660 (54.4)	1323 (54.1)	1337 (54.7)	
55-69	2645 (31.6)	1031 (32.9)	1614 (30.8)		1544 (31.6)	789 (32.3)	755 (30.9)	
>=70	585 (7.0)	224 (7.1)	361 (6.9)		344 (7.0)	158 (6.5)	186 (7.6)	
Marital status				0.094				0.426
Married	5004 (59.7)	1915 (61.1)	3089 (58.9)		3015 (61.7)	1529 (62.5)	1486 (60.8)	
Single	1748 (20.9)	619 (19.8)	1129 (21.5)		961 (19.7)	473 (19.3)	488 (20.0)	
Other	1625 (19.4)	600 (19.1)	1025 (19.5)		914 (18.7)	443 (18.1)	471 (19.3)	
Race				0.127				0.305
White	5914 (70.6)	2225 (71.0)	3689 (70.4)		3576 (73.1)	1806 (73.9)	1770 (72.4)	
Black	1266 (15.1)	444 (14.2)	822 (15.7)		670 (13.7)	335 (13.7)	335 (13.7)	
Other	1197 (14.3)	465 (14.8)	732 (14.0)		644 (13.2)	304 (12.4)	340 (13.9)	
Laterality				0.013				0.391
Left	4230 (50.5)	1638 (52.3)	2592 (49.4)		2541 (52.0)	1286 (52.6)	1255 (51.3)	
Right	4147 (49.5)	1496 (47.7)	2651 (50.6)		2349 (48.0)	1159 (47.4)	1190 (48.7)	
Grade				<0.001				0.535
I-II	1622 (19.4)	489 (15.6)	1133 (21.6)		830 (17.0)	408 (16.7)	422 (17.3)	
III-IV	2568 (30.7)	827 (26.4)	1741 (33.2)		1417 (29.0)	696 (28.5)	721 (29.5)	
Unknown	4187 (50.0)	1818 (58.0)	2369 (45.2)		2643 (54.0)	1341 (54.8)	1302 (53.3)	
T stage				0.003				0.993
T1	2043 (24.4)	798 (25.5)	1245 (23.7)		1172 (24.0)	585 (23.9)	587 (24.0)	
T2	4665 (55.7)	1771 (56.5)	2894 (55.2)		2831 (57.9)	1415 (57.9)	1416 (57.9)	
T3	1669 (19.9)	565 (18.0)	1104 (21.1)		887 (18.1)	445 (18.2)	442 (18.1)	
ER status				0.023				0.640
Positive	5163 (61.6)	1882 (60.1)	3281 (62.6)		2953 (60.4)	1485 (60.7)	1468 (60.0)	
Negative	3214 (38.4)	1252 (39.9)	1962 (37.4)		1937 (39.6)	960 (39.3)	977 (40.0)	
PR status				0.003				0.668
Positive	4057 (48.4)	1451 (46.3)	2606 (49.7)		2356 (48.2)	1170 (47.9)	1186 (48.5)	
Negative	4320 (51.6)	1683 (53.7)	2637 (50.3)		2534 (51.8)	1275 (52.1)	1259 (51.5)	
HER2 status				<0.001				0.062
Positive	3206 (38.3)	1277 (40.7)	1929 (36.8)		1945 (39.8)	940 (38.4)	1005 (41.1)	
Negative	5171 (61.7)	1857 (59.3)	3314 (63.2)		2945 (60.2)	1505 (61.6)	1440 (58.9)	
Breast subtype				<0.001				0.291
HR+/HER2+	2124 (25.4)	847 (27.0)	1277 (24.4)		1286 (26.3)	620 (25.4)	666 (27.2)	
HR+/HER2-	3240 (38.7)	1112 (35.5)	2128 (40.6)		1776 (36.3)	912 (37.3)	864 (35.3)	
HR-/HER2+	1082 (12.9)	430 (13.7)	652 (12.4)		659 (13.5)	320 (13.1)	339 (13.9)	
HR-/HER2-	1931 (23.1)	745 (23.8)	1186 (22.6)		1169 (23.9)	593 (24.3)	576 (23.6)	
Breast surgery				<0.001				0.300
BCS	3340 (39.9)	1528 (48.8)	1812 (34.6)		2147 (43.9)	1055 (43.1)	1092 (44.7)	
MAST	5037 (60.1)	1606 (51.2)	3431 (65.4)		2743 (56.1)	1390 (56.9)	1353 (55.3)	
Radiation				0.057				0.502
YES	6192 (73.9)	2354 (75.1)	3838 (73.2)		3723 (76.1)	1851 (75.7)	1872 (76.6)	
NO	2185 (26.1)	780 (24.9)	1405 (26.8)		1167 (23.9)	594 (24.3)	573 (23.4)	

PSM, propensity-score matching; SLNB, sentinel lymph node biopsy; ALND, axillary lymph node dissection; ER, estrogen receptor; PR, progesterone receptor; HER2, human epidermal growth factor receptor 2; HR, hormone receptor; BCS, breast-conserving surgery; MAST, Mastectomy.

### Survival analysis

**Figure 2:**
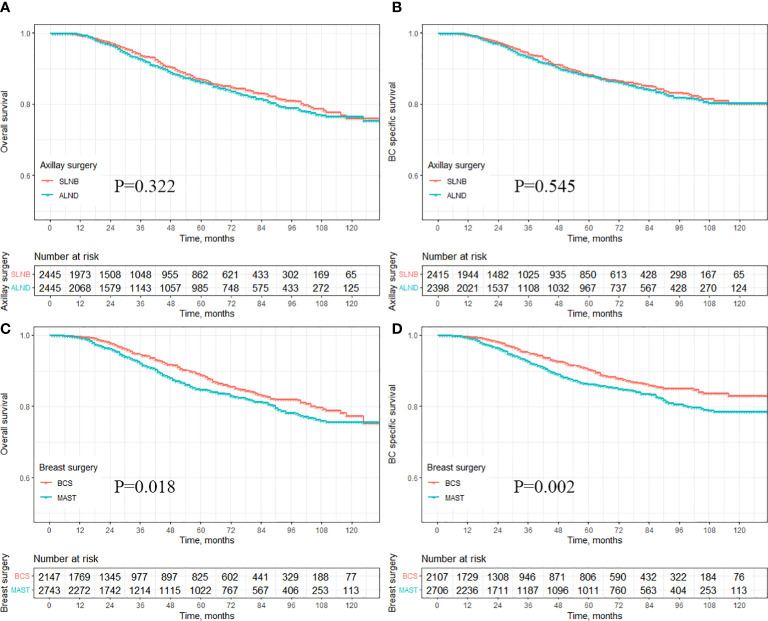
Survival analysis of different axillary and breast surgeries for OS and BCSS of breast cancer patients. **(A)** OS in the axillary surgery group, **(B)** BCSS in the axillary surgery group, **(C)** OS in the breast surgery group, **(D)** BCSS in the breast surgery group.

During a median follow-up period of 32 months (ranging, 1-131 months), there were 215 deaths in the SLNB group, of which 185 (86.0%) were attributed to breast cancer. In the ALND group, there were 266 deaths, with 219 (82.3%) being due to breast cancer. Kaplan-Meier survival analysis revealed that for cT1-3N1M0 breast cancer patients, those treated with ALND demonstrated slightly lower OS and BCSS compared to those treated with SLNB. However, these differences did not reach statistical significance (HR=1.10, 95% CI 0.91-1.31, P=0.322 vs. HR=1.06, 95% CI 0.87-1.29, P=0.545) (**Figures 2A, B**). When comparing the MAST group to the BCS group, patients in the MAST group exhibited significantly worse OS and BCSS (HR=1.25, 95% CI 1.04-1.50, P=0.018 vs. HR=1.37, 95% CI 1.12-1.68, P=0.002) (**Figures 2C, D**).

**Figure 3:**
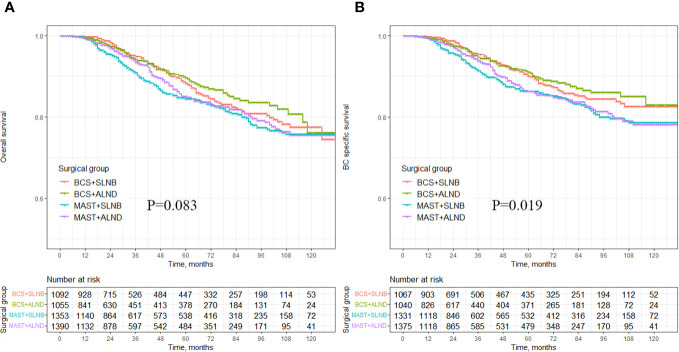
Survival analysis of OS and BCSS in breast cancer patients with different axillary surgery combined with different breast surgery. **(A)** OS in the different axillary surgery combined with different breast surgery, **(B)** BCSS in the different axillary surgery combined with different breast surgery.

There was no statistically significant difference in the impact of different combinations of axillary and breast surgeries (BCS+SLNB, BCS+ALND, MAST+SLNB, and MAST+ALND) on OS (*p* = 0.083) (**Figure 3A**). However, after excluding breast cancer-related deaths caused by other factors, it was observed that various combinations of axillary and breast surgeries did have a significant effect on BCSS. Specifically, MAST combined with ALND showed the poorest BCSS and this difference was statistically significant (*p* = 0.019) (**Figure 3B**). Additionally, the use of BCS+SLNB in combination with radiotherapy was associated with improved OS in cT1-3N1M0 breast cancer patients (*p* = 0.038) (**Supplementary Figure S1A**). Both BCS+SLNB and ALND combined with radiotherapy demonstrated improvements in BCSS (*p* = 0.042, *p* = 0.031) (**Supplementary Figure S1B**).

### Univariate and multivariate Cox regression analysis

**Figure 4:**
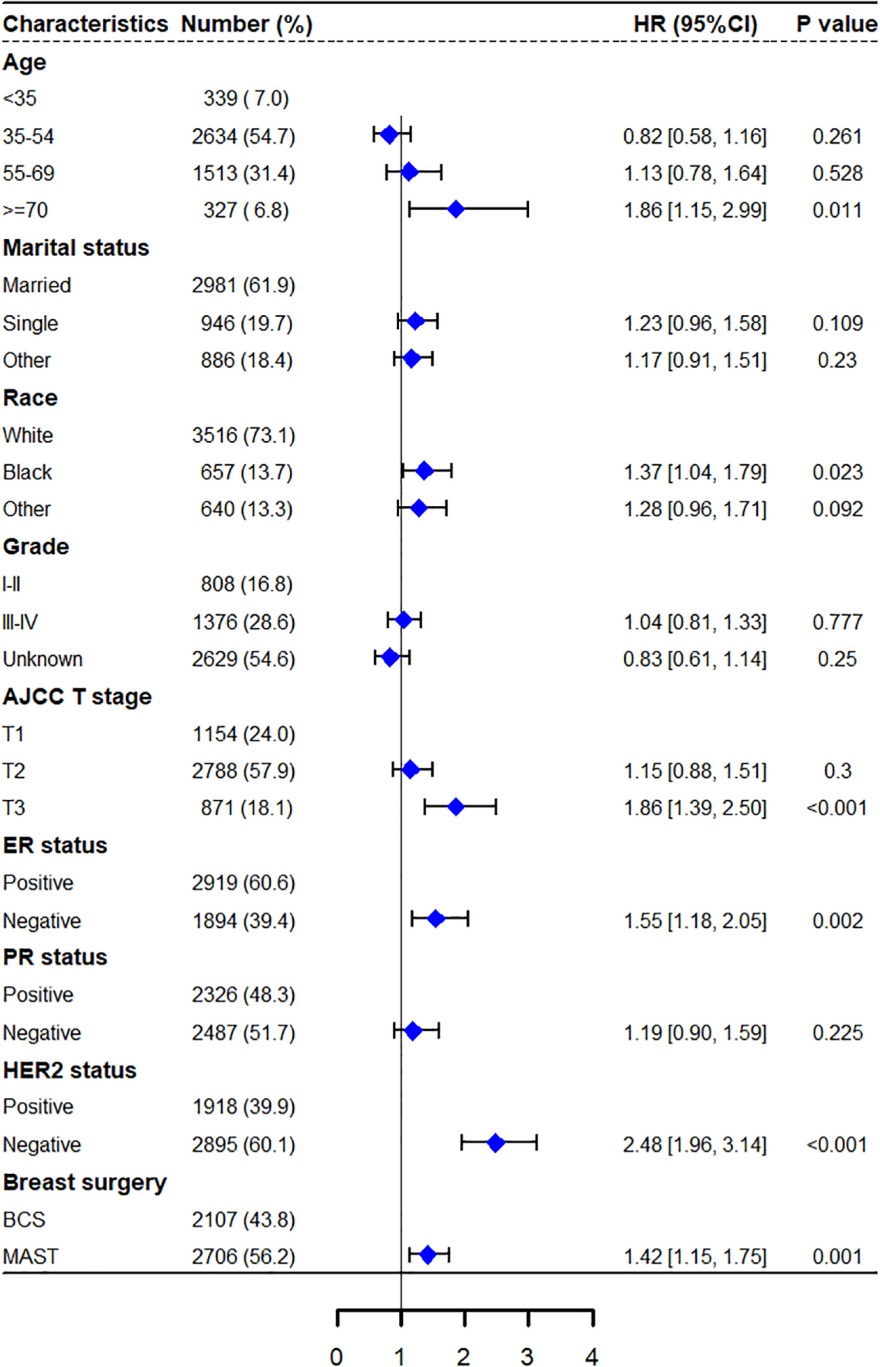
Multivariate Cox regression model forest graph for BCSS.

In the univariate Cox regression analysis, various factors including different age groups, marital status, race, histological grade, T stage, ER expression, PR expression, HER2 expression, molecular typing, and type of breast surgery were found to be significantly correlated with both OS and BCSS, establishing them as independent prognostic predictors (all *p* < 0.05). However, laterality, axillary surgery, and radiotherapy were not found to be associated with OS and BCSS (all *p* > 0.05) (**Table 2**). Following the removal of two collinear variables (molecular subtypes and combined axillary operation with breast operation), statistically significant variables identified in the univariate analysis were included in the multivariate Cox proportional risk regression model analysis, and a forest plot model was constructed. The results indicated that age ≥ 70, being of black race, T3 staging, ER-negative expression, HER2 negative expression, and undergoing mastectomy were identified as independent prognostic risk factors for both OS and BCSS, with all differences being statistically significant (all *p* < 0.05) (**Table 3**). Furthermore, having a marital status categorized as “other” emerged as an independent prognostic factor for overall survival (HR=1.27, 95% CI 1.01-1.59, p = 0.040). The forest plots depicting the results of the multivariate Cox regression models for both BCSS and OS can be found in **Figure 4** and **Supplementary Figure S2**, respectively.

**Table 2:** Univariate Cox prognostic analysis of OS and BCSS.

Characteristics	OS		BCSS	
	HR[95% CI]	P value	HR[95% CI]	P value
Age				
<35	Reference		Reference	
35-54	0.71 (0.51-0.98)	0.036	0.68 (0.48-0.95)	0.024
55-69	0.95 (0.68-1.32)	0.742	0.84 (0.59-1.2)	0.339
>=70	1.8 (1.2-2.69)	0.004	1.39 (0.89-2.19)	0.148
Marital status				
Married	Reference		Reference	
Single	1.3 (1.04-1.63)	0.023	1.3 (1.02-1.65)	0.037
Other	1.54 (1.23-1.91)	<0.001	1.38 (1.08-1.77)	0.010
Race				
White	Reference		Reference	
Black	1.52 (1.2-1.93)	0.001	1.56 (1.21-2.03)	0.001
Other	1.07 (0.81-1.41)	0.627	1.2 (0.9-1.6)	0.216
Laterality				
Left	Reference		Reference	
Right	1.13 (0.94-1.35)	0.181	1.15 (0.95-1.4)	0.157
Grade				
I-II	Reference		Reference	
III-IV	1.28 (1.04-1.59)	0.021	1.31 (1.04-1.66)	0.022
Unknown	1.04 (0.79-1.38)	0.759	1.02 (0.75-1.38)	0.922
T stage				
T1	Reference		Reference	
T2	1.1 (0.87-1.4)	0.416	1.16 (0.89-1.52)	0.271
T3	1.76 (1.35-2.29)	<0.001	1.96 (1.46-2.62)	<0.001
ER status				
Postive	Reference		Reference	
Negative	1.8 (1.51-2.16)	<0.001	1.81 (1.49-2.2)	<0.001
PR status				
Postive	Reference		Reference	
Negative	1.65 (1.37-1.98)	<0.001	1.69 (1.38-2.07)	<0.001
HER2 status				
Postive	Reference		Reference	
Negative	2.18 (1.77-2.69)	<0.001	2.46 (1.94-3.11)	<0.001
Breast subtype				
HR+/HER2+	Reference		Reference	
HR+/HER2-	2.17 (1.61-2.93)	<0.001	2.35 (1.69-3.27)	<0.001
HR-/HER2+	1.77 (1.23-2.54)	0.002	1.64 (1.08-2.48)	0.020
HR-/HER2-	3.9 (2.9-5.25)	<0.001	4.23 (3.05-5.87)	<0.001
Breast surgery				
BCS	Reference		Reference	
MAST	1.25 (1.04-1.5)	0.019	1.37 (1.12-1.68)	0.002
Axillay surgery				
SLNB	Reference		Reference	
ALND	1.1 (0.91-1.31)	0.322	1.06 (0.87-1.29)	0.545
Radiation				
YES	Reference		Reference	
NO	0.98 (0.8-1.2)	0.851	1.02 (0.81-1.27)	0.889
Surgical group				
BCS+ALND	Reference		Reference	
BCS+SLNB	0.89 (0.67-1.19)	0.444	0.93 (0.67-1.29)	0.668
MAST+ALND	1.23 (0.97-1.58)	0.092	1.37 (1.04-1.8)	0.023
MAST+SLNB	1.13 (0.88-1.46)	0.335	1.28 (0.97-1.7)	0.080

OS, overall survival; BCSS, breast cancer-specific survival; HR, hazard ratio; ER, estrogen receptor; PR, progesterone receptor; HER2, human epidermal growth factor receptor 2; HR, hormone receptor; BCS, breast-conserving surgery; MAST, Mastectomy; SLNB, sentinel lymph node biopsy; ALND, axillary lymph node dissection.

**Table 3:** Multivariate Cox prognostic analysis of OS and BCSS.

Characteristics	OS		BCSS	
	HR[95% CI]	P value	HR[95% CI]	P value
Age				
<35	Reference		Reference	
35-54	0.83(0.6-1.16)	0.277	0.82(0.58-1.16)	0.261
55-69	1.19(0.84-1.7)	0.332	1.13(0.78-1.64)	0.528
>=70	2.25(1.46-3.45)	<0.001	1.86(1.15-2.99)	0.011
Marital status				
Married	Reference		Reference	
Single	1.24(0.99-1.57)	0.065	1.23(0.96-1.58)	0.109
Other	1.27(1.01-1.59)	0.040	1.17(0.91-1.51)	0.230
Race				
White	Reference		Reference	
Black	1.36(1.06-1.74)	0.015	1.37(1.04-1.79)	0.023
Other	1.17(0.88-1.54)	0.278	1.28(0.96-1.71)	0.092
Grade				
I-II	Reference		Reference	
III-IV	1.03(0.82-1.3)	0.778	1.04(0.81-1.33)	0.777
Unknown	0.86(0.65-1.15)	0.311	0.83(0.61-1.14)	0.250
T stage				
T1	Reference		Reference	
T2	1.13(0.89-1.44)	0.326	1.15(0.88-1.51)	0.300
T3	1.74(1.33-2.28)	<0.001	1.86(1.39-2.5)	<0.001
ER status				
Postive	Reference		Reference	
Negative	1.61(1.25-2.08)	<0.001	1.55(1.18-2.05)	0.002
PR status				
Postive	Reference		Reference	
Negative	1.12(0.86-1.46)	0.403	1.19(0.9-1.59)	0.225
HER2 status				
Postive	Reference		Reference	
Negative	2.19(1.77-2.7)	<0.001	2.48(1.96-3.14)	<0.001
Breast surgery				
BCS	Reference		Reference	
MAST	1.32(1.09-1.59)	0.005	1.42(1.15-1.75)	0.001

OS, overall survival; BCSS, breast cancer-specific survival; HR, hazard ratio; ER, estrogen receptor; PR, progesterone receptor; HER2, human epidermal growth factor receptor 2; BCS, breast-conserving surgery; MAST, Mastectomy.

### Competing risk model analysis

**Figure 5:**
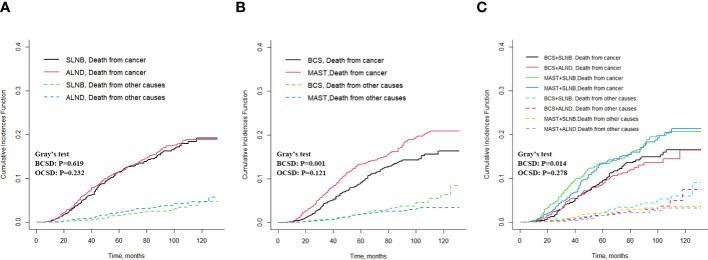
Cumulative incidence of BCSD and OCSD for different axillary and breast surgeries. **(A)** BCSD and OCSD in the axillary surgery group, **(B)** BCSD and OCSD in the breast surgery group, **(C)** BCSD and OCSD in the different axillary surgery combined with different breast surgery.

To mitigate the influence of non-breast cancer-related deaths on survival analysis, a competitive risk model was employed for the analysis. The results of the Fine-Gray test indicated no significant difference in the cumulative incidence of BCSD (Grey’s test, *p* = 0.619) and OCSD (Grey’s test, P=0.232) between the ALND and SLNB groups (**Figure 5A**). When comparing the MAST group to the BCS group, the cumulative incidence of BCSD was notably higher (Grey’s test, *p* = 0.001), signifying a statistically significant difference. However, the cumulative incidence of OCSD in the MAST group did not show statistical significance (Grey’s test, P=0.121) (**Figure 5B**). Furthermore, in comparison to the combination of BCS with SLNB or ALND, the MAST group combined with SLNB or ALND exhibited a significantly higher cumulative incidence of BCSD (Grey’s test, *p* = 0.014), while the cumulative incidence of OCSD was not statistically significant (Grey’s test, *p* = 0.278) (**Figure 5C**). The multivariate analysis conducted with the competitive risk model identified age ≥ 70, being of black race, T3 staging, ER-negative expression, HER2-negative expression, and undergoing mastectomy as independent prognostic risk factors, all demonstrating statistically significant differences (all *p* < 0.05) (**Supplementary Table S1**). The nomogram illustrating the competitive risk model is presented in **Supplementary Figure S3**.

## Discussion

BCS combined with SLNB has been performed in cN1+ breast cancer patients effectively treated with NAT. This approach has been a subject of ongoing debate in clinical practice, particularly due to the limited evidence on long-term survival outcomes from extensive real-world data. In this retrospective study, we analyzed data from 8377 patients diagnosed with non-specific infiltrating duct carcinoma of cT1-3N1M0 breast cancer in the SEER database between 2010 and 2020. After meticulous matching using PSM to minimize confounding bias, a total of 4890 patients were included in the final analysis. The results revealed that the benefits of SLNB on both OS and BCSS were comparable to those of ALND. Moreover, patients who underwent BCS demonstrated significantly better OS and BCSS compared to those who underwent MAST. Additionally, combining BCS with either SLNB or ALND led to improved survival outcomes. We further employed Fine-Gray competitive risk analysis and Cox proportional risk regression models to account for the impact of deaths from other causes on survival outcomes. These analyses revealed a higher cumulative incidence of BCSD in patients who underwent MAST combined with either SLNB or ALND. Based on our findings, we recommend the combination of BCS and SLNB for patients who meet the criteria for breast and axillary preservation.

In this study, it was observed that 37.4% of patients with cN1+ breast cancer underwent SLNB. Existing literature reports a wide range of SLNB proportions in cN1+ breast cancer patients effectively treated with NAT, varying from 14.6% to 56.5%. Simultaneously, the rate of ALND decreased from 100% to 29.4% (11, 13, 17). Various factors have been associated with the reduction in ALND rates after NAT, including breast cancer molecular subtype (11, 13), higher histological grade (11), residual breast lesions, and vascular infiltration (11, 19). However, Weber et al. presented data indicating that the ALND rate in cN1+ breast cancer patients who effectively responded to NAT remained as high as 49% Interestingly, their study found no correlation between the acceptance of ALND and the proportion or treatment regimen of adjuvant therapy after NAT (27).

SLN staging after NAT has demonstrated greater accuracy in reflecting prognosis compared to the initial axillary status. Most studies support the implementation of SLNB after NAT (1, 13, 15, 19, 21). It is recommended to utilize the dual tracer method or positioning clip for marking positive lymph nodes, with SLN detection rates ranging from 80.1% to 96%, and false negative rates from 6.8% to 17% (1, 12, 16, 19–24). To further minimize the false negative rate, axillary lymph nodes can be labeled with radioactive iodine seeds, resulting in detection rates of SLN ranging from 98.2% to 100%, false negative rates of 2-4%, negative prediction rates of 92-97%, and an 82% reduction in the need for ALND. However, the practice of implanting guidewires under ultrasound guidance to locate suspicious lymph nodes before NAT is not recommended, as it yields a detection rate of only 70.8% (24, 28–30). Additionally, in order to decrease the false negative rate of detected SLNs, it is recommended to increase the number of SLNs examined and employ immunohistochemical techniques. With three or more SLNs examined, the false negative rate is notably low, ranging from 0-9% (11, 12, 16, 21, 23).

In this study, we observed no significant difference in OS and BCSS between the SLNB group and the ALND group. This finding aligns with the results reported in the majority of literature. For instance, Martelli et al. demonstrated that in cT2N0/1 breast cancer patients receiving NAT, the 10-year OS in the SLNB group was 89% with a 10-year Disease-Free Survival (DFS) of 79%, showing no significant difference in survival outcomes compared to the SLNB+ALND group (14). Similarly, Kahler-Ribeiro-Fontana et al. found that cN1+ breast cancer patients who underwent SLNB after NAT exhibited a 5-year OS rate of 89.8% and a 10-year OS rate of 80.1% (31). In a study by Kim et al., N+ breast cancer patients who received NAT were stratified into five groups based on surgical approach and pathological axillary lymph node results, revealing no disparities in OS or axillary local recurrence rate among the groups (20). Moreover, Piltin et al. reported that among breast cancer patients who underwent SLNB after NAT and were followed for a median of 34 months, recurrence occurred in only 1 out of 159 patients, in contrast to 16 out of 443 patients who underwent ALND (17).

In this study, we observed that nearly 44% of patients with cN1+ breast cancer opted for BCS, resulting in an improved appearance and enhanced psychological well-being for these patients. After NAT, the rate of breast preservation in patients has shown a consistent upward trend, reaching 53.2% to 90% (8–10). BCS is deemed feasible even for patients with multifocal or multicentric lesions, provided there is no residual tumor at the surgical margin. Studies have demonstrated that there are no significant differences in local recurrence, disease-free survival, and overall survival when the surgical margin exceeds 2mm or 1mm, as compared to margins less than 2mm or 1mm (32, 33). The success of transitioning to BCS is associated with factors like the molecular subtype of breast cancer, larger tumor size, positive axillary lymph nodes, and the presence of breast calcification (9). Among breast cancer patients who underwent breast-preserving surgery following NAT, the 10-year local recurrence rate in the breast was 6.5%, while the 10-year recurrence rate in the axillary region of the breast was 10.3%. In comparison to mastectomy, there were no statistically significant differences in terms of distant recurrence, BCSD, and OCSD, although the local recurrence rate was slightly higher. High local recurrence was associated with ER-negativity, cN1+ status, non-pathological complete response in axillary lesions, and pN2-3 staging. To mitigate the risk of local recurrence, it is imperative to implement measures such as meticulous local and pathological evaluation, precise tumor localization, intraoperative removal of breast markers, accurate determination of the volume of the lesion to be resected, and the consideration of adjuvant radiotherapy (7, 34). Sang et al. corroborated that following NAT, breast cancer patients who underwent BCS exhibited a significantly improved overall survival rate compared to those who opted for mastectomy. This finding aligns with the conclusions drawn in the present study, where no statistically significant disparities were observed in terms of disease-free survival and local recurrence between the two groups (10).

The study results indicate that combining BCS with SLNB or ALND leads to improved survival outcomes. Additionally, the inclusion of postoperative radiotherapy to both the breast and axillary regions is recommended to further enhance these outcomes (1, 2, 6, 15). In this study, cN1+ breast cancer patients who responded effectively to NAT and underwent BCS in combination with SLNB demonstrated significantly improved OS and BCSS benefits after receiving postoperative supplemental radiation therapy. It’s worth mentioning that Rusthoven et al.’s findings suggested that, following mastectomy after NAT, radiotherapy improved OS across all postoperative axillary lymph node subgroups (ypN0, ypN1, and ypN2-3). Interestingly, in patients undergoing BCS, regardless of axillary lymph node status, radiotherapy to the whole breast and regional lymph nodes did not lead to improved OS, which contrasts with the conclusions of this study (35). In line with the majority of literature, this study identified age ≥ 70, black race, T3 stage, ER-negative expression, and HER2-negative status as independent prognostic risk factors for BCSS, further corroborating existing evidence.

This study benefits from an extensive dataset comprising nearly 510,000 patients of breast cancer over an 11-year period, sourced from the SEER database. PSM analysis was effectively utilized to mitigate potential confounding variables, enhancing the robustness of the conclusions. The extended follow-up period of more than 10 years from the date of diagnosis further strengthens the reliability of the findings. However, the study does possess certain limitations. Firstly, it is a retrospective study without a predefined experimental design, resulting in the absence of specific variables related to axillary surgery methods, such as SLNB procedure codes, number of SLNs detected, SLN tracing methods, and precise chemoradiotherapy protocols. This could introduce bias and limits further in-depth analysis. Secondly, despite the study’s extended duration, the median follow-up time of 32 months suggests that a majority of enrolled cases are recent, potentially resulting in fewer recorded death events and influencing the analysis of survival outcomes to some degree. Finally, various factors impacting survival outcomes, including targeted medications, endocrine treatments, genetic testing, and underlying patient conditions, are not included in the SEER database, preventing further analysis. Despite these constraints, the study’s findings still offer valuable evidence for guiding the selection of axillary breast surgery for breast cancer patients who respond effectively to NAT. Nevertheless, confirmation through large-scale, multi-center prospective cohort studies is warranted.

## Conclusion

Utilizing SEER data, we investigated the prognostic implications of distinct axillary and breast surgical approaches in cT1-3N1M0 breast cancer patients exhibiting responsiveness to NAT. Among cN1+ breast cancer patients effectively treated with NAT, the combined approach of BCS and SLNB emerged as the optimal surgical strategy for those meeting criteria for axillary and breast-sparing surgery. This approach demonstrated superior long-term quality of life and survival outcomes.

## Data availability statement

The datasets presented in this study can be found in online repositories. The names of the repository/repositories and accession number(s) can be found in the article/**Supplementary Material**.

## Author contributions

MZ: Data curation, Investigation, Writing – review & editing. YS: Conceptualization, Data curation, Writing – review & editing. HSW: Data curation, Formal analysis, Writing – review & editing. JX: Investigation, Methodology, Writing – review & editing. WC: Investigation, Methodology, Writing – review & editing. HW: Validation, Visualization, Writing – review & editing. BY: Validation, Visualization, Writing – review & editing. HL: Writing – review & editing, Validation, Visualization.

## Supplementary material

Supplementary Figure S1 Survival analysis of breast cancer patients treated with different axillary and breast surgeries combined with radiotherapy.

Supplementary Figure S2 Multivariate Cox regression model forest graph for OS.

Supplementary Figure S3 Nomogram of multivariate competitive risk regression model analysis.
