# Supplementary figures and images for: Prognostic analysis of cT1-3N1M0 breast cancer patients who have responded to neoadjuvant therapy undergoing various axillary surgery and breast surgery based on propensity score matching and competitive risk model
Source: Front Oncol. 2024 Jan 24;14:1319981. doi: 10.3389/fonc.2024.1319981 (PMC10847357; doi:10.3389/fonc.2024.1319981)

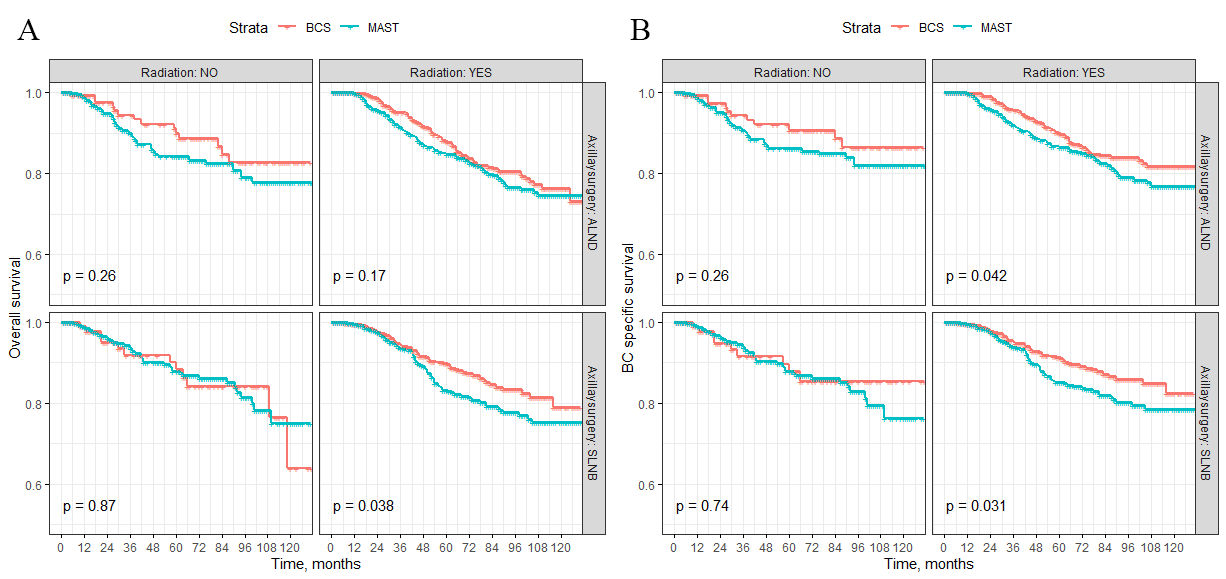

Supplement: Supplementary Figure S1 — Survival analysis of breast cancer patients treated with different axillary and breast surgeries combined with radiotherapy. [file Image_1.tiff]

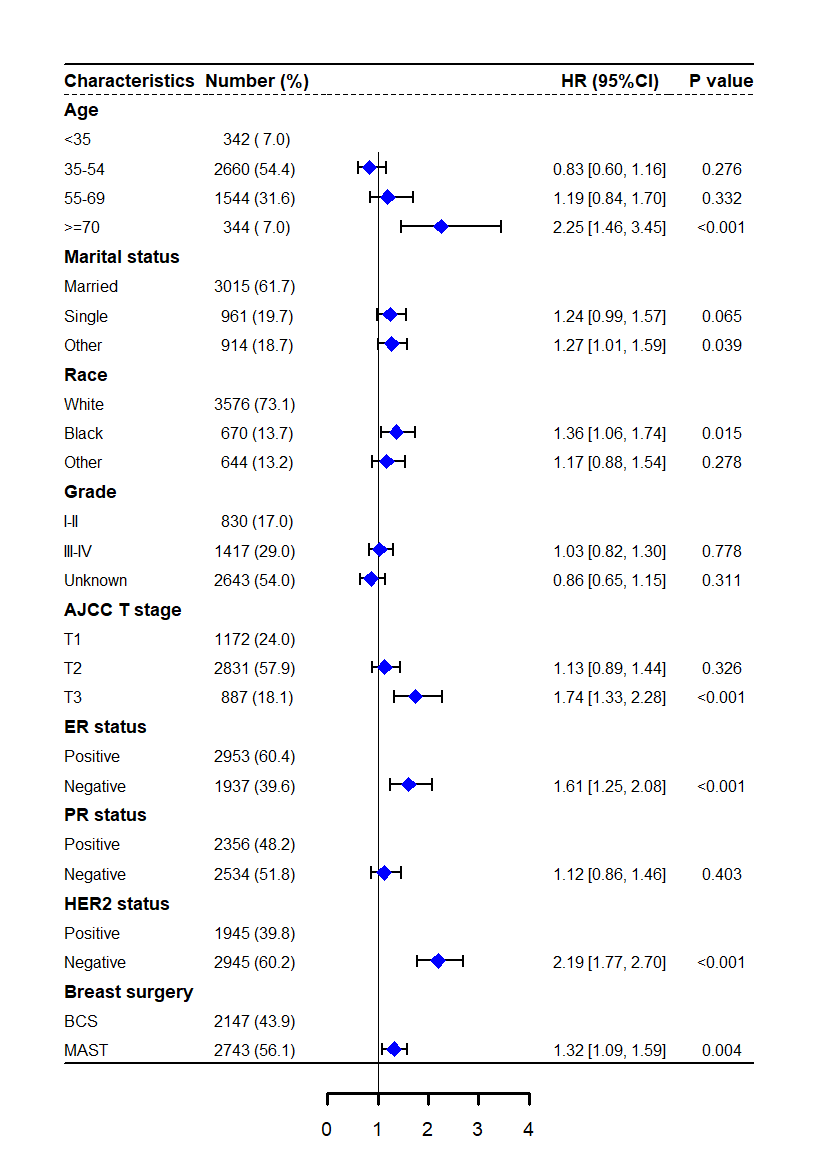

Supplement: Supplementary Figure S2 — Multivariate Cox regression model forest graph for OS. [file Image_2.tiff]

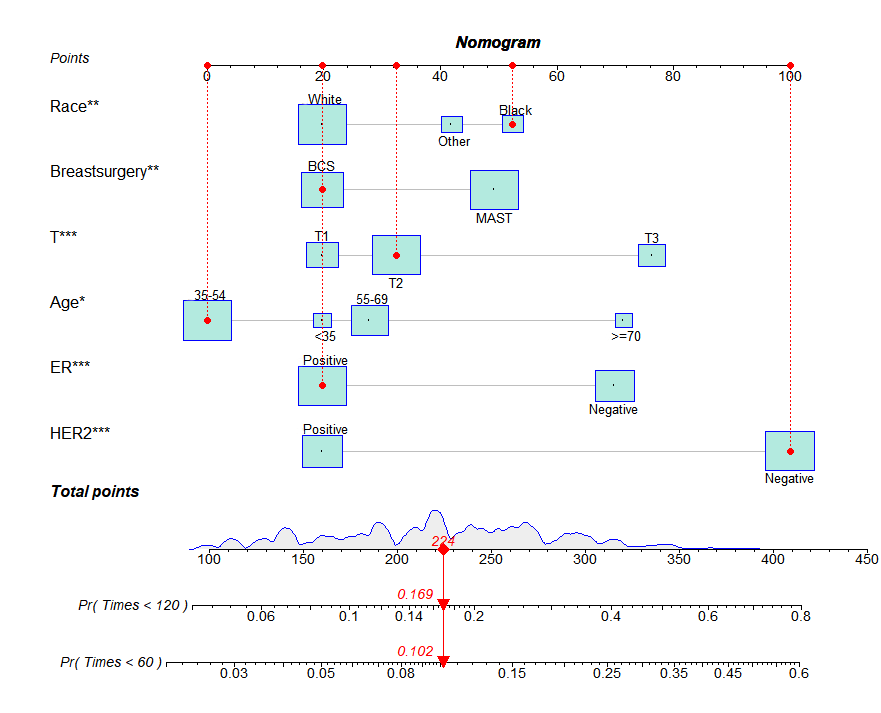

Supplement: Supplementary Figure S3 — Nomogram of multivariate competitive risk regression model analysis. [file Image_3.tiff]
