# Supplementary material for: Prognostic analysis of cT1-3N1M0 breast cancer patients who have responded to neoadjuvant therapy undergoing various axillary surgery and breast surgery based on propensity score matching and competitive risk model
Source: Front Oncol. 2024 Jan 24;14:1319981. doi: 10.3389/fonc.2024.1319981 (PMC10847357; doi:10.3389/fonc.2024.1319981)
Supplement: Supplementary file 4 [file Table_1.pdf]

TABLE S1 Multivariate competitive risk regression model analysis

| Characteristics | HR[95% CI] | P value |
|---|---|---|
| Age | | |
| <35 | Reference | |
| 35-54 | 0.80(0.57-1.13) | 0.201 |
| 55-69 | 1.10(0.76-1.58) | 0.620 |
| >=70 | 1.80(1.13-2.84) | 0.012 |
| Race | | |
| White | Reference | |
| Black | 1.45(1.11-1.88) | 0.006 |
| Other | 1.29(0.96-1.72) | 0.088 |
| T stage | | |
| T1 | Reference | |
| T2 | 1.15(0.88-1.51) | 0.292 |
| T3 | 1.90(1.41-2.55) | <0.001 |
| ER status | | |
| Postive | Reference | |
| Negative | 1.76(1.45-2.15) | <0.001 |
| HER2 status | | |
| Postive | Reference | |
| Negative | 2.48(1.96-3.14) | <0.001 |
| Breast surgery | | |
| BCS | Reference | |
| MAST | 1.40(1.13-1.72) | 0.002 |

HR, hazard ratio; ER, estrogen receptor; HER2, human epidermal growth factor receptor 2; BCS, breast-conserving surgery; MAST, Mastectomy.
